# Everolimus versus alpelisib in advanced hormone receptor-positive HER2-negative breast cancer: targeting different nodes of the PI3K/AKT/mTORC1 pathway with different clinical implications

**DOI:** 10.1186/s13058-020-01271-0

**Published:** 2020-04-06

**Authors:** Claudio Vernieri, Francesca Corti, Federico Nichetti, Francesca Ligorio, Sara Manglaviti, Emma Zattarin, Carmen G. Rea, Giuseppe Capri, Giulia V. Bianchi, Filippo de Braud

**Affiliations:** 1 IFOM, the FIRC Institute of Molecular Oncology, Via Adamello 16, 20139 Milan, Italy; 2 Medical Oncology Department, Fondazione IRCCS Istituto Nazionale dei Tumori, Via Venezian, 1, 20133 Milan, Italy; 3 Oncology and Hemato-Oncology Department, University of Milan, 20122 Milan, Italy

**Keywords:** Advanced breast cancer, Hormone receptor-positive breast cancer, Endocrine therapy, mTORC1, PI3K, Everolimus, Alpelisib, *PIK3CA* mutations, Efficacy comparisons

## Abstract

**Background:**

The PI3K/AKT/mTORC1 axis is implicated in hormone receptor-positive HER2-negative metastatic breast cancer (HR+ HER2− mBC) resistance to anti-estrogen treatments. Based on results of the BOLERO-2 trial, the mTORC1 inhibitor everolimus in combination with the steroidal aromatase inhibitor (AI) exemestane has become a standard treatment for patients with HR+ HER2− mBC resistant to prior non-steroidal AI therapy. In the recent SOLAR-1 trial, the inhibitor of the PI3K alpha subunit (p110α) alpelisib in combination with fulvestrant prolonged progression-free survival (PFS) when compared to fulvestrant alone in patients with *PIK3CA-*mutated HR+ HER2− mBC that progressed after/on previous AI treatment. Therefore, two different molecules targeting the PI3K/AKT/mTORC1 axis, namely everolimus and alpelisib, are available for patients progressing on/after previous AI treatment, but it is unclear how to optimize their use in the clinical practice.

**Main body of the abstract:**

Here, we reviewed the available clinical evidence deriving from the BOLERO-2 and SOLAR-1 trials to compare efficacy and safety profiles of everolimus and alpelisib in advanced HR+ HER2− BC treatment. Adding either compound to standard endocrine therapy provided similar absolute and relative PFS advantage. In the SOLAR-1 trial, a 76% incidence of grade (G) 3 or 4 (G3/G4) adverse events was reported, while G3/G4 toxicities occurred in 42% of patients in the BOLERO-2 trial. While alpelisib was only effective in patients with *PIK3CA-*mutated neoplasms, retrospective analyses indicate that everolimus improves exemestane efficacy independently of *PIK3CA* mutational status.

**Conclusions:**

Based on the available efficacy and safety data, the “new” alpelisib may be burdened by higher incidence of severe adverse events, higher costs, and anticancer efficacy that is limited to *PIK3CA-*mutated tumors when compared to the “old” everolimus. Therefore, the everolimus-exemestane combination remains an effective and reasonably well-tolerated therapeutic option for HR+ HER2− mBC patients progressing after/on previous AI treatment, independently of *PIK3CA* mutational status.

## Background

Endocrine therapy (ET) is the mainstay of treatment for patients with hormone receptor-positive (HR+) human epidermal growth factor receptor 2-negative (HER2−) metastatic breast cancer (mBC) [1]. However, tumors initially responding to ET, including the most recent ET-Cyclin-Dependent Kinase 4/6 (CDK4/6) inhibitor combinations, almost invariably develop resistance [2–4]. Hence, the identification of targeted therapies that are able to revert or delay endocrine resistance is a clinically relevant issue.

Aberrant signaling through the phosphatidylinositol 3-kinase/protein kinase B (AKT)/mechanistic target of rapamycin complex 1 (PI3K/AKT/mTORC1) cascade is clearly implicated in endocrine resistance, thus providing the rationale for combining inhibitors of this pathway with currently available ET [5–7]. Based on the results of the BOLERO-2 trial, the mTORC1 inhibitor everolimus (Eve) has been approved in combination with the aromatase inhibitor (AI) exemestane (Exe) for the treatment of HR+ HER2− mBC progressing on/after one line of non-steroidal aromatase inhibitor (NSAI) treatment [8]. More recently, the PI3Kα-specific inhibitor alpelisib (Alp) plus fulvestrant (Fulv) combination significantly prolonged progression-free survival (PFS) when compared to Fulv alone in patients with *PIK3CA*-mutated HR+ HER2− mBC, thus leading to FDA registration of Alp in this clinical setting [9]. Based on results of the SOLAR-1 study, Alp is increasingly considered by treating physicians and experts in the field as a candidate to replace Eve in HR+ HER2− mBC treatment [10].

Here, we review data from prospective trials to compare the antitumor efficacy and safety profile of Eve/ET and Alp/ET combinations in women with HR+ HER2− mBC. We also discuss how Alp and Eve could fit in the future treatment scenario of mBC.

## Main text

### The biology of the PI3K/AKT/mTORC1 axis

The insulin receptor (IR)/PI3K/AKT/mTORC1 pathway is the most commonly dysregulated pathway in human cancers and plays a crucial role in stimulating tumor cell metabolism, growth, proliferation, and motility [11]. PI3Ks include three classes of kinases with different structural properties and biological functions. Among different PI3Ks, class I PI3Ks, which include class IA (p110α, p110β, and p110δ) and class IB (p110γ) PI3Ks, have been found to be more commonly dysregulated in human cancers [11]. Enhanced activation of the IR/PI3K/AKT/mTORC1 axis can result from (a) increased extracellular concentration of growth factors activating oncogenic receptor tyrosine kinases (RTKs), such as IR or insulin-like growth factor 1 (IGF-1) receptor (IGF1R), on cell plasma membranes [12]; (b) activating mutations or overexpression of RTKs, including members of the HER family for class IA PI3Ks, or G protein-coupled receptors (GPCR) for class IB PI3Ks [13]; and (c) activating mutations or overexpression of downstream kinases, such as PI3K subunits, AKT and mTORC1, or inactivation of the phosphatase and tensin homolog deleted from chromosome 10 (PTEN), tuberous sclerosis complex 1/2 (TSC1/2), or liver kinase B1 (LKB1) tumor suppressor proteins [11].

**Fig. 1:**
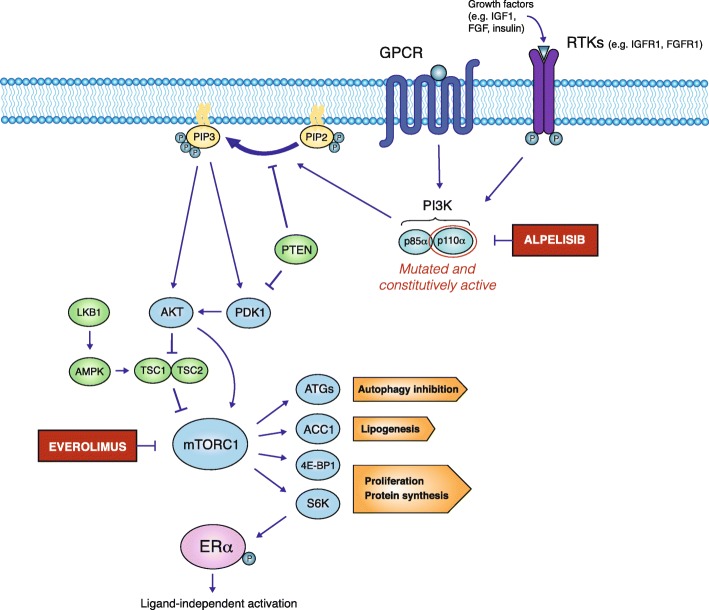
Schematic representation of the PI3K/AKT/mTOR axis and its alterations in breast cancer. Alpelisib selectively inhibits the p110α subunit of PI3K, which is mutated and constitutively activated in approximately 40% of HR+ HER2− BC

Once activated by upstream signals, the PI3K regulatory subunit p85α binds to the phospho-tyrosine residues on receptor protein kinases or adaptor proteins, such as insulin receptor substrate 1 (IRS1), and unleashes the PI3K catalytic subunit p110α (encoded by the *PIK3CA* gene), which is enabled to phosphorylate phosphatidylinositol 4,5-bisphosphate (PIP2) to phosphatidylinositol 3,4,5-triphosphate (PIP3) (Fig. 1) [14, 15]. On the other hand, mutated (i.e., constitutively active) PI3K subunits catalyze PIP3 biosynthesis independently of upstream signals; in particular, mutations of the *PIK3CA* gene are found in approximately 40% of HR+ HER2− BCs and cause constitutive PI3K activation [16, 17]. Once synthesized, PIP3 anchors the serine/threonine AKT kinase to the cell plasma membrane, where it activates mTORC1, either directly or through the inhibition of TSC1/TSC2 [11, 13, 17]. In turn, mTORC1 stimulates cell growth and proliferation by triggering protein translation initiation through phosphorylating eIF4E-binding proteins (4E-BPs) and S6 kinases (S6K1 and S6K2). mTORC1 also inhibits autophagy and stimulates lipogenesis via intermediate lipogenic transcriptional factors and mitochondrial biogenesis (Fig. 1). Overall, mTORC1 activation induces a global metabolic response leading to the stimulation of anabolic processes and macromolecule biosynthesis [18, 19].

In parallel with mTORC1 activation, constitutively active PI3K stimulates several biological processes that stimulate tumor cell proliferation, such as the Mitogen Activated Protein Kinase (MAPK) and estrogen receptor α (ERα) pathways [20], as well as the reprogramming of glucose and lipid metabolism via AKT activation and AMPK inhibition (Fig. 1) [15, 21].

Tumor suppressor enzymes prevent uncontrolled activation of the PI3K/AKT/mTORC1 cascade at different levels: among them, PTEN counteracts PI3K activity by dephosphorylating PIP3 to PIP2, while LKB1 indirectly inhibits mTORC1 via AMP-activated protein kinase (AMPK)-mediated activation of TSC1/2 [22, 23].

Notably, the PI3K/AKT/mTORC1 pathway is aberrantly activated in approximately 70% of BCs as a result of increased extracellular concentration of growth factors, activating mutations of genes encoding RTKs (e.g., IGFR1 and fibroblast growth factor receptor 1 [FGFR1]) or downstream oncogenes (e.g., *PI3KCA* or *AKT*), or, finally, loss-of-function or reduced levels of PTEN, LKB1, or inositol polyphosphate 4-phosphatase type II (INPP4B) tumor suppressor proteins [24, 25]. Among these alterations, *PIK3CA* mutations are by far the most common ones [16]. Oncogenic *PIK3CA* mutations include the following: the kinase domain H1047R mutation (exon 20), which results in higher binding affinity of PI3K to the plasma membrane and to PIP2; the helical domain E542K and E545K mutations (exon 9), which enable the direct interaction of PI3K catalytic subunit with IRS1 independently of p85 and IRS1 phosphorylation; and deletions in the C2 domain, which unleash inhibitory contacts with regulatory subunits [13, 14].

Alp selectively binds to and inhibits p110α, while Eve inhibits mTORC1 downstream of PI3K through allosteric binding. When used in in vitro models of HR+ HER2− BC, both PI3K and mTORC1 inhibitors demonstrated synergistic anticancer activity in combination with anti-estrogens. For instance, HR+ BC cells treated with letrozole (Let) plus Eve accumulate in the G1 phase of the cell cycle and undergo proliferation inhibition and apoptosis [26, 27]. Moreover, the mTORC1 inhibitor rapamycin reverts resistance to Fulv or tamoxifen (TAM) in HR+ BC cell lines, both alone and in combination with ET [27]. Finally, inhibitors of p110α and/or p110β showed synthetic lethal effects when combined with different ETs [5, 28, 29]. Mechanistically, these synergistic effects are the result of a crosstalk between the PI3K/AKT/mTORC1 and ER signaling pathways. One of mTORC1 targets, S6K1, is responsible for N-terminal ERα Activation Function 1 (AF1) domain phosphorylation on Serine167, thus leading to its ligand-independent transactivation [20]. Therefore, S6K-induced, ligand-independent activation of ERα can induce HR+ BC resistance to ET, thus providing strong preclinical rationale for combining PI3K/AKT/mTORC1 pathway inhibitors with ET to prevent/revert endocrine resistance.

Since Eve inhibits the PI3K/AKT/mTORC1 cascade downstream of PI3K, its antitumor activity should be independent of *PIK3CA* mutational status. Conversely, Alp selectively inhibits proliferation of PI3Kα-driven HR+ HER2− BC cells and causes regression of *PIK3CA*-mutated in vivo tumor models [6, 28]. Therefore, *PIK3CA-*mutated tumors are the best candidates to respond to Alp [30].

### BOLERO-2 and SOLAR-1 trials: a comparison of efficacy and safety data

The BOLERO-2 and SOLAR-1 studies are the two randomized trials leading to Eve and Alp registration for HR+ HER2− mBC treatment in combination with standard ET. The main clinical and tumor characteristics of patients enrolled in the BOLERO-2 and SOLAR-1 (only *PIK3CA-*mutated cohort) trials are summarized in Table 1.

**Table 1:** Clinical and tumor characteristics in patients enrolled in the BOLERO-2 and SOLAR-1 trials (cohort of *PIK3CA-*mutated cancers)

Patient/tumor characteristic	Everolimus and exemestane group (*N* = 485)	Placebo and exemestane group (*N* = 239)	Alpelisib and fulvestrant group (*N* = 169)	Placebo and fulvestrant group (*N* = 172)
Age (years)				
Median	62	61	63	64
Range	34–93	28–90	25–87	38–92
ECOG Performance Status^a^ (%)				
0	60	59	66.3	65.7
1	36	35	33.1	33.7
2	2	3	0	0
Missing data	NA	NA	0.6	0.6
Visceral disease (%)	56	56	55	58.1
Metastatic site (%)				
Lung	29	33	33.7	39.5
Liver	33	30	29	31.4
Bone	76	77	NA	NA
No. of metastatic sites (%)				
0–1	32	29	37.3	30.2
2	31	34	34.3	34.9
≥ 3	36	37	28.4	34.3
Previous chemotherapy (%)				
Neoadjuvant or adjuvant only	44	40	59.8	62.2
Treatment of metastatic disease (with or without neoadjuvant or adjuvant therapy)	26	26	0	0.6
Previous CDK 4/6 inhibitors^b^ (%)	0	0	5.3	6.4

^a^*ECOG Performance Status:* Eastern Cooperative Oncology Group Performance Status

^b^*CDK 4/6* : Cyclin-Dependent Kinase 4/6

The BOLERO-2 was a double-blind, phase III study that investigated the efficacy of the Eve/Exe combination in HR+ HER2− mBC postmenopausal women previously treated with NSAIs [8]. Patients (*n* = 724) enrolled in the trial were randomized in a 2:1 ratio to receive Eve/Exe or placebo/Exe. The primary endpoint was PFS; secondary endpoints were overall survival (OS), overall response rate (ORR), and safety. Median PFS was 11.0 months in the experimental arm versus (vs.) 4.1 months in the control arm (hazard ratio [HR] 0.38; 95% confidence interval [CI] 0.31–0.48; *p* < 0.0001; Table 2), with an ORR of 12.6% vs. 2.1%, respectively [31]. No significant differences in terms of OS were observed between Eve/Exe and placebo/Exe (median 31.0 months vs. 26.6 months, respectively; HR 0.89; 95% CI 0.73–1.10; *p* = 0.14) [32]. As for the safety profile, severe (G3/G4) AEs occurred in 33% and 9% of patients receiving the experimental or standard treatment, respectively, with stomatitis (8% vs. < 1%), anemia (6% vs. < 1%), dyspnea (4% vs. 1%), hyperglycemia (6% vs. 1%), fatigue (5% vs. 1%), and pneumonitis (4% vs. 0%) being the most common ones [8, 33] (Table 3). Median duration of Eve treatment was 5.5 months, with the main cause of therapy discontinuation being disease progression (61.9% vs. 88.7% in the Eve and control arms, respectively), followed by AEs (26.3% vs. 5%, respectively). Notably, next-generation sequencing (NGS) analysis performed in archival tumor specimens from a subgroup (*n* = 302) of patients enrolled in the BOLERO-2 trial showed that Eve provides clinical benefit to patients with both *PIK3CA*-wild type (wt) (HR 0.37; 95% CI 0.25–0.55) and *PIK3CA*-mutated (HR 0.51; 95% CI 0.34–0.77) tumors [34].

**Table 2:** Efficacy analysis data from the BOLERO-2 trial and the SOLAR-1 study (cohort of PIK3CA-mutated cancer)

Clinical endpoint	Everolimus and exemestane group (*N* = 482)	Placebo and exemestane group (*N* = 238)	Alpelisib and fulvestrant group (*N* = 169)	Placebo and fulvestrant group (*N* = 172)
Best overall response (%)				
Complete response CR	0	0	0.6	1.2
Partial response PR	12.6	2.1	26.0	11.6
Stable disease SD	73.4	62.8	34.3	36.6
Neither complete response nor progressive disease^a^	/	/	22.5	14.5
Progressive disease PD	5.8	23.4	9.5	30.8
Unknown	8.2	11.7	7.1	5.2
Overall response (%)	12.6	2.1	26.6	12.8
Clinical benefit (%)	49.9	22.2	61.5	45.3

“/,” not evaluated in this trial

^a^In this category, the best overall response was evaluated only in patients who had no measurable disease at baseline according to the Response Evaluation Criteria in Solid Tumors, version 1.1

**Table 3:** Incidence of adverse events in different arms in the BOLERO-2 and SOLAR-1 trials

Adverse event	Everolimus and exemestane group (*N* = 482)			Placebo and exemestane group (*N* = 238)			Alpelisib and fulvestrant group (*N* = 284)			Placebo and fulvestrant group (*N* = 287)		
	Any grade	Grade 3	Grade 4	Any grade	Grade 3	Grade 4	Any grade	Grade 3	Grade 4	Any grade	Grade 3	Grade 4
Hyperglicemia	16	6	< 1	3	1	0	63.7	32.7	3.9	9.8	0.3	0.3
Stomatitis	67	8	0	12	< 1	0	24.6	2.5	0	6.3	0	0
Rash	36	1	0	6	0	0	35.6	9.9	0	5.9	0.3	0
Fatigue	37	4	< 1	27	1	0	24.3	3.5	0	17.1	1.0	0
Diarrhea	30	2	< 1	16	1	0	57.7	6.7	0	15.7	0.3	0
Nausea	27	< 1	< 1	27	1	0	44.7	2.5	0	22.3	0.3	0
Decreased appetite	29	1	0	10	0	0	35.6	0.7	0	10.5	0.3	0
Vomiting	14	< 1	< 1	11	< 1	0	27.1	0.7	0	9.8	0.3	0
Weight loss	19	1	0	5	0	0	26.8	3.9	0	2.1	0	0
Dysgeusia	21	< 1	0	5	0	0	16.5	0	0	3.5	0	0
Headache	19	< 1	0	13	0	0	17.6	0.7	0	13.2	0	0
Asthenia	12	2	0	3	0	0	20.4	1.8	0	12.9	0	0
Pruritus	11	< 1	0	3	0	0	18	0.7	0	5.6	0	0
Arthralgia	16	1	0	16	0	0	11.3	0.4	0	16.4	1.0	0
Cough	22	1	0	11	0	0	/	/	/	/	/	/
Dyspnea	18	4	0	9	1	< 1	/	/	/	/	/	/
Pneumonitis	12	3	0	0	0	0	/	/	/	/	/	/
Anemia	16	5	1	4	< 1	< 1	/	/	/	/	/	/
Thrombocytopenia	12	2	1	< 1	0	< 1	/	/	/	/	/	/
Epistaxis	15	0	0	1	0	0	/	/	/	/	/	/
Pyrexia	14	< 1	0	6	< 1	0	/	/	/	/	/	/
Peripheral edema	14	1	0	6	< 1	0	/	/	/	/	/	/
AST level increased^a^	13	3	< 1	6	1	0	/	/	/	/	/	/
ALT level increased^b^	11	3	< 1	3	2	0	/	/	/	/	/	/
Constipation	13	< 1	0	11	< 1	0	/	/	/	/	/	/
Insomnia	11	< 1	0	8	0	0	/	/	/	/	/	/
Back pain	11	0	0	8	1	0	/	/	/	/	/	/
Hyperlipidemia	14	1	0	2	0	0	/	/	/	/	/	/
Infections and infestations	50	5	2	25	2	0	/	/	/	/	/	/
Alopecia	/	/	/	/	/	/	19.7	0	0	2.4	0	0
Mucosal inflammation	/	/	/	/	/	/	18.3	2.1	0	1.0	0	0

“/,” adverse event not evaluated in this trial

^a^Aspartate aminotransferase

^b^Alanine aminotransferase

The SOLAR-1 study was a double-blind, phase III trial that randomized 571 postmenopausal women (*n* = 571; 99.83%) or men (*n* = 1; 0.17%) previously treated with an AI to receive Alp plus Fulv or placebo plus Fulv [9]. The determination of *PIK3CA* gene mutational status in tumor tissue specimens was mandatory before patient enrollment. Indeed, based on *PIK3CA* status (mutated vs. wt), patients were assigned to two different cohorts; then, they were randomized in a 1:1 ratio to receive the experimental (Alp/Fulv) or standard (placebo/Fulv) treatment. The primary endpoint of the SOLAR-1 trial was PFS in the cohort of *PIK3CA*-mutated patients, i.e., those patients with the highest chances to benefit from the experimental treatment based on previous preclinical and clinical studies [30, 35]. Secondary endpoints included OS in the cohort of patients with *PIK3CA*-mutated tumors, PFS and OS in the *PIK3CA*-wt cohort, ORR, clinical benefit, and treatment safety in the whole patient population. Notably, less than 7% of patients in all treatment arms had received previous treatment with CDK4/6 inhibitors. After a median follow-up of 20 months, median PFS for patients in the *PIK3CA*-mutated cohort was 11 months in the experimental arm vs. 5.7 months in the control arm (HR 0.65; 95% CI 0.50–0.85; *p* < 0.0001 Table 2), with an ORR of 26.6% and 12.8%, respectively. In the cohort of patients with *PIK3CA*-wt tumors, the experimental treatment was associated with a non-significant difference in terms of median PFS (7.4 vs. 5.6 months, respectively; HR 0.85; 95% CI 0.58–1.25). G3 and G4 AEs occurred in 64.4% and 11.6%, respectively, of Alp/Fulv-treated patients, and in 30.3% and 5.2%, respectively, of placebo/Fulv-treated subjects. The most common G3/G4 AEs in the experimental arm were hyperglycemia (36.6%), rash (9.9%), and diarrhea (6.7%). With a median duration of exposure to Alp of 5.5 months, the most frequent reasons of treatment discontinuation were disease progression (55% vs. 68% in the Alp/Fulv and placebo/Fulv groups, respectively) and the occurrence of AEs (25% vs. 4.2%, respectively), with hyperglycemia and rash being the most common AEs leading to permanent treatment discontinuation (Table 3). Regarding Alp-induced hyperglycemia, patients with fasting plasma glucose levels equal to 140 mg/dl or higher than 140 mg/dl received metformin as per SOLAR-1 protocol. Therefore, metformin administration was started before patients developed grade 3 or 4 hyperglycemia (fasting plasma glucose levels > 250 mg/dl) in most of the cases. Despite this practice, the incidence of severe hyperglycemia in Alp-treated patients was 36.6%, and it reasonable to speculate that it might have been even superior without the precocious administration of metformin [36].

Even if the BOLERO-2 and SOLAR-1 trials enrolled patients with overall similar characteristics at baseline (Table 1), some differences need to be highlighted: (a) the BOLERO-2 trial enrolled patients with ECOG PS of 0–2, while the SOLAR-1 trial only enrolled patients with an ECOG PS of 0–1; (b) patients with previously treated and stable brain metastases were included in the SOLAR-1, but not in the BOLERO-2 trial; (c) enrollment of male patients was allowed in the SOLAR-1, but not in the BOLERO-2 trial; however, only one male patient was finally enrolled in the SOLAR-1 study; (d) patients with type 1 or uncontrolled type 2 diabetes mellitus were excluded from the SOLAR-1 but not from the BOLERO-2 trial; (e) a higher percentage of patients in the SOLAR-1 trial (52.1% in the *PIK3CA-*mutated cohort) received Alp/Fulv as their first-line treatment for advanced disease when compared to patients treated with Eve/Exe in the BOLERO-2 study (20.6%) [37]; (f) 26% of patients treated with Eve/Exe in the BOLERO-2 trial had received previous chemotherapy for the treatment of advanced disease, whereas these patients were excluded from the SOLAR-1 study; (g) 11.8% of patients with *PIK3CA-*mutated tumors treated with Alp/Fulv in the SOLAR-1 trial had endocrine-sensitive disease, which was an exclusion criterion in the BOLERO-2 trial; and (h) the type of ET combined with the experimental drug was different in the two studies (Exe and Fulv, respectively).

Except for the inclusion of patients with brain metastases, the SOLAR-1 trial enrolled a more selected population of HR+ HER2− mBC patients with less pretreated and potentially more endocrine-sensitive disease. This could at least in part explain the longer PFS observed in patients in the control arm of the SOLAR-1 trial (5.7 months) when compared to patients in the control arm of the BOLERO-2 study (4.1 months in the overall population; 2.8 months in a subgroup of patients with *PIK3CA-*mutated tumors [34]). Despite these differences, the absolute PFS advantage provided by the addition of Eve or Alp to standard ET was similar (6.9 and 5.3 months, respectively, when considering the whole population of patients enrolled in the BOLERO-2 trial and patients with *PIK3CA-*mutated neoplasms in the SOLAR-1 study; 3.9 and 5.3 months, respectively, when considering only patients with *PIK3CA-*mutated tumors in both studies). The relative PFS advantage associated with Eve (HR 0.36) was higher than the relative benefit associated with Alp (HR 0.65) when considering all patients enrolled in the BOLERO-2 trial, while it was similar in subgroups of patients with *PIK3CA-*mutated tumors (0.51 and 0.65, respectively) [34]. The rate of treatment discontinuation was high in both studies (about 25%), but the incidence of G3/G4 AEs was considerably higher in both the treatment (76% vs. 42%, respectively) and control (35.5% vs. 9%, respectively) arm of the SOLAR-1 trial.

### Other prospective studies investigating Eve or Alp

After the publication of the BOLERO-2 study, other prospective phase IIIb–IV trials (4EVER [38], BRAWO [39], STEPAUT [40], BALLET [41], EVEREXES [42]) investigated the efficacy and tolerability of Eve/Exe in more heterogeneous patient cohorts when compared to patients enrolled in the BOLERO-2 trial (Table 4) [8]. In particular, the 4EVER, BRAWO, and BALLET studies enrolled patients independently of the number of previous chemotherapy lines for advanced disease, as well as of previous Exe treatment, thus more faithfully recapitulating patients treated in the real-world clinical practice [38, 39, 41]. For instance, 60% and 53.7% of patients in the BALLET and 4EVER studies, respectively, had received previous chemotherapy for advanced disease when compared to 26% of patients in the BOLERO-2 study. Nonetheless, activity and efficacy data from these studies were similar to those from the BOLERO-2 trial, with ORR ranging from 8.2% (BRAWO) to 15.8% (EVEREXES), and mPFS ranging from 5.6 months (4EVER) to 9.5 months (STEPAUT, EVEREXES). The safety profile of Eve/Exe was also consistent with data from the BOLERO-2 study, with the most commonly observed G3/G4 toxicities being stomatitis (range 3.9–10.6%), dyspnea (range 2–4.7%), asthenia/fatigue (range 1.5–3.6%), and hyperglycemia (range 2.9–7%). Treatment discontinuation rates due to AEs ranged from 17.1% (BALLET) to 26% (BRAWO). While the safety profile of Eve/Exe in elderly patients (> 70 years) in the BALLET study was overall similar to that observed in the BOLERO-2 trial, incidence of G3/G4 AEs, dose reductions/interruptions, and treatment discontinuations due to AEs were higher in the elderly vs. non-elderly population [41].

**Table 4:** Efficacy and safety data from Eve prospective studies published after the BOLERO-2 trial in HR+, HER2− aBC/mBC

Everolimus											
Study	Study design	Population	N° of pts.	Previous CT allowed	Median TD (mos)/(R)DI^a^ (mg/d)	ORR	mPFS (mos)	mOS (mos)	Any grade AEs (%) (Eve combination)^b^	G3/4 AEs (%) (Eve combination) ^b^	Discontinuation rate^c^
4EVER [38], phase IIIb, open label, single arm	**Eve + Exe** (10 + 25 mg/d)	Postmenopausal HR+, HER2− LABC/mBC progressing on or after an NSAI (either adjuvant or for advanced disease)	299^b^	Yes, any number of lines for LABC/mBC, prior Exe allowed	**TD/RDI**, 4.4/0.98	8.9% (at 24 weeks)	5.6	mOS NR, OS at 48w 66.9%	Overall 98.7% Stomatitis 49.2% Fatigue 36.1% Diarrhea 26.4% Nausea 26.1%	Overall 58.9% Stomatitis 8.4% GPHD 6.7% Dyspnea 4.7% Anemia 4.3%	24.7%
BRAWO [39], phase IV, non-interventional	**Eve + Exe** (5–10 + 25 mg/d)	HR+, HER2− LABC/mBC progressed after a NSAI Eve + Exe as per clinical practice	2074	Yes, previous Exe allowed	**TD 10 mg/d**, 5.1 **TD 5 mg/d**, 4.6	8.2%	6.6	NA	Stomatitis 42.6% Fatigue 19.8%	Stomatitis 3.9% Fatigue 1.5%	26%
STEPAUT [40], phase IV, non-interventional	**Eve + Exe** (5–10 + 25 mg/d)	Postmenopausal HR+, HER2− LABC/mBC progressing on/after prior NSAIs in routine clinical practice	225	NS	**TD/DI**, NA/NA	NA	9.5	NA	Stomatitis/mucositis 48% Rash/exanthema 22.2% Dyspnea/cough 22.2%	Stomatitis/mucositis 4.4% GPHD/weight loss 2.7% Inappetence /nausea 2.2%	NA
BALLET [41], phase IIIb, open label, single arm, expanded access trial	**Eve + Exe** (5–10 + 25 mg/d)	Postmenopausal HR+, HER2− LABC/mBC progressing on/after prior NSAIs	2133	Yes, any number of lines for LABC/mBC	**TD/RDI**, 3.7/0.98	NA	NA	NA	Overall 94.7% Stomatitis 52.8% Asthenia 22.8% Diarrhea 16.8% Rash 16.5% Inappetence 16%	Overall 42.7% Stomatitis 9.4% Asthenia 3.6% Hyperglycemia 2.9% Dyspnea 2% NIP 1.9%	17.1%
EVEREXES [42], phase IIIb, open label, single arm, Asia and Africa	**Eve + Exe** (10 + 25 mg/d)	Postmenopausal HR+, HER2− LABC/mBC progressing on/after prior NSAI (adjuvant or for LABC/mBC)	232	Yes, no more than 1 prior CT line for LABC/mBC	**TD/DI**, NA/9.2	15.8%	9.5	NA	Stomatitis 60.4% Skin toxicity 27.8% Hyperglycemia 24.7% Fatigue 17.2% Weight loss 15.4%	Stomatitis 10.6% Hyperglycemia 7% Fatigue 2.2% NIP 1.3% Weight loss 0.9%	NA
BOLERO-4 [43], phase II, multicenter, open-label, single-arm	First line: **Eve + Let** (10 + 2.5 mg/d); at PD second line: **Eve + Exe** (10 + 25 mg/d)	Postmenopausal HR+ HER2− LABC/mBC	202, 50	No	**TD/DI**, 14.8/8.5 and 2.9/8.3	45%, 6%	22, 3.7	mOS NR, OS at 24 m 78.7%	**Eve + Let** Overall 100% Stomatitis 68.8% Loss of weight 44% Diarrhea 41% Nausea 37%	**Eve + Let** Overall 58% Anemia 10% Hypertension 8% Stomatitis 6% Hypertriglyceridemia 6%	**Eve + Let** 15.8%
TAMRAD [44], phase II, open-label, randomized	**Eve + TAM** (10 + 20 mg/d) **vs. TAM** (20 mg/d)	Postmenopausal HR+, HER2−, LABC/mBC progressing on/after prior NSAI (adjuvant or for LABC/mBC)	54, 57	Yes, any number of lines for LABC/mBC	**TD/DI**, 6.2/NA and 4.8/NA	14%, 13%	8.6, 4.5	NR, 32.9	Pain 82% Fatigue 72% Anemia 69% Stomatitis 56% Leukopenia 54%	Stomatitis 11% Pain 9% Infections 7% Anorexia 7% Fatigue 6%	**Eve + TAM** 22%
PrE0102 [45], phase II, randomized, double-blind, placebo-controlled	**Eve + Fulv**^**e**^ (10 mg/d)^f^**vs. Fulv**^**e**^	Postmenopausal HR+ HER2− LABC/mBC progressing on/after prior NSAI (adjuvant or for LABC/mBC)	66, 65	Yes, no more than 1 prior CT line for LABC/mBC	**TD/DI**, 5.1/NA and 4.6/NA	18.2%, 12.3%	10.3, 5.1	28.3, 31.4	Mucositis 53% Fatigue 42% Rash 38% Anemia 31% Diarrhea 23%	Mucositis 11% NIP 6% Fatigue 6%	**Eve + Fulv** 20%
MANTA [46], phase II, open-label, randomized	**Eve + Fulv**^**e**^ (10 mg/d)^f^, **cVIS + Fulv**^**e**^, (50 mg BID)^g^, **iVIS + Fulv**^**e**^ (2 days on, 5 days off; 125 mg BID)^h^, **Fulv**^**e**^	Postmenopausal HR+, LABC/mBC progressing on/after prior NSAI (either adjuvant or for LABC/mBC)	65, 103, 98, 67	Yes, no more than 1 prior CT line for LABC/mBC	**TD/DI**, NA/NA for all arms	41.2%, 30.4%, 28.6%, 25.0%	12.3, 7.6, 8.0, 5.4	NR, 27.1, 24.2, 24.4	Stomatitis 60% Asthenia 53.3% Rash 50.0% Diarrhea 31.7% Decreased appetite 30.0%	Stomatitis 11.7% Rash 5.0% Asthenia 3.3% Diarrhea 1.7% Decreased appetite 1.7%	18.8%
Safra et al. [47], phase II, open-label, single-arm, multicenter trial	**Eve + Let** (10 + 2.5 mg/d)	Postmenopausal ER+, HER2− LABC/mBC progressing on/after prior ET (either adjuvant or for LABC/mBC)	72	No	**TD/DI**, NA/NA	23.3%	8.8	22.9	Fatigue 61.1% Stomatitis 54.2% Rash 33.4% Cough 33.3% Decreased appetite 31.9%	Anemia 9.7% Stomatitis 8.3% Fatigue 5.6% Diarrhea 5.6% Hyperglycemia 4.2%	12.5%
BOLERO-6 [48], phase II, open-label, randomized	**Eve + Exe** (10 + 25 mg/d) **vs. Eve** (10 mg/d) **vs. capecitabine** (1250 mg/m2 BID)	Postmenopausal HR+ HER2− LABC/mBC progressing on/after prior NSAI	104, 103, 102	Yes, no more than 1 prior CT line for LABC/mBC, prior Exe not allowed	**TD/RDI**, 6.3/0.92, 4.6/0.98, and 6.1/0.78	NA	8.4, 6.8, 9.6	23.1, 29.3, 25.6	Overall 100% Stomatitis 49% Fatigue 38% Diarrhea 35% Anemia 32% GGT elevation 15% AST elevation 15%	Overall 70% Anemia 13% Stomatitis 9% GGT elevation 9% Fatigue 8% AST elevation 7% Pneumonitis 7%	**Eve + Exe** 8%
Yardley et al. [49], phase II, open label	**Eve** (10 mg/d) added to the most recent **ET** on which a patient progressed	Post/premenopausal HR+, HER2− LABC/mBC refractory to ET (either adjuvant or for LABC/mBC)	47	Yes no more than 1 prior CT line for LABC/mBC	**TD/DI**, 4.1/NA	6%	6.6	21.1	Fatigue 38% Stomatitis 32% Mucosal inflammation 28% Rash 28%	Fatigue 4% Stomatitis 6% Mucosal inflammation 4% Rash 4%	15%

*AEs* adverse events, *AST* aspartate aminotransferase, *BID* bis in die, *CT* chemotherapy, *cVIS* continuous vistusertib, *d* day, *Eve* everolimus, *Exe* exemestane, *ET* endocrine therapy, *Fulv* fulvestrant, *G* grade, *GGT* gamma glutamyl transferase, *GHPD* general physical health deterioration, *mos* months, *HER2* human epidermal growth factor receptor 2, *HR* hormone receptor, *iVIS* intermittent vistusertib, *LABC* locally advanced breast cancer, *Let* letrozole, *mBC* metastatic breast cancer, *mg/d* milligrams per day, *mPFS* median progression-free survival, *mOS* median overall survival, *N*° number, *NA* not available, *NE* not evaluable, *NIP* non-infectious pneumonitis, *NR* not reached, *NS* not specified, *NSAI* non-steroideal aromatase inhibitor, *ORR* overall response rate, *PD* progressive disease, *pts.* patients, (*R)DI* (relative) dose intensity, *TAM* tamoxifen, *TD* treatment duration, *w* weeks

^a^Only absolute but not relative dose intensity is calculated in mg/d

^b^Reported AEs refer only to the arm including Eve + ET

^c^Study treatment discontinuation (referring to the arm containing Eve + ET) due to AEs

^d^281 patients evaluable for efficacy, 299 patients for safety

^e^Fulv 500 mg intramuscular injection on day 1, followed by 500 mg doses on days 15 and 28, and then every 28 days

^f^Refers to Eve

^g^Refers to cVIS

^h^Refers to iVIS

Altogether, real-world data corroborate the efficacy of Eve in combination with ET for the treatment of HR+ HER2− mBC. Subgroup analyses of these studies indicate that ORR and PFS may be lower in patients treated with a higher number of previous therapy lines, with previous exposure to chemotherapy, or treated with lower Eve treatment intensity [38–40]. Finally, no ORR or PFS differences have been described based on prior treatment with Exe [38]. Of note, the introduction of prophylactic dexamethasone oral solution for the prevention or management of Eve-induced stomatitis has remarkably improved the safety profile of Eve through reducing one of the most common and disturbing toxicities related to the use of this compound [50].

More recently, the phase II BOLERO-4 study evaluated Eve plus Let as a first-line treatment in 202 postmenopausal women with HR+ HER2 mBC, who received second-line Eve/Exe on progression. First-line Eve/Let was associated with mPFS of 22.0 months (95% CI 18.1–25.1), while mOS was not reached. Of note, mPFS was 3.7 months (95% CI 1.9–7.4 months) with second-line Eve/Exe treatment (50 patients) [43]. While these data indicate that Eve/Let is an effective first-line combination treatment, they also show that Eve continuation after disease progression is a poorly effective therapeutic strategy. Other phase II studies evaluating Eve in combination with Let, Fulv, Exe, or TAM in patients with mBC progressing on/after prior NSAI therapy showed interesting activity and efficacy, in the absence of relevant unforetold toxicities [44–49].

As for Alp, small prospective trials published before the SOLAR-1 study evaluated the Let/Alp or Fulv/Alp combinations in patients with HR+ HER2− mBC progressing after previous ET. Consistent with SOLAR-1 results, these studies reported an incidence of G3/G4 hyperglycemia and rash in the 10–38.1% and 8–27.8% ranges, respectively, with longer mPFS in patients with *PIK3CA-*mutated neoplasms (Table 5) [30, 35, 51].

**Table 5:** Efficacy and safety data from phase Ib/II trials of alpelisib in HR+, HER2− aBC/mBC

Alpelisib										
Study	Study design	Population	N° of pts.	Previous CT allowed	ORR	mPFS (mos)	mOS (mos)	Any grade AEs (%)	G3/4 AEs (%)	Discontinuation rate^a^
Juric et al. [30], phase Ib, open-label, single-arm	**Alpelisib + Fulv**^**b**^ (300–350–400 mg/d)^d^	Postmenopausal *PIK3CA*-mutated (60%) or *PIK3CA*-wt (38%) HR+, LABC/mBC progressing on/after prior ET	87	NS	*PIK3CA*-mutated, 29%; *PIK3CA*-wt, 0%	*PIK3CA*-mutated, 9.1; *PIK3CA*-wt, 4.7	NA	Diarrhea 60% Nausea 53% Hyperglycemia 51%	Hyperglycemia 22% Maculopapular rash 13% Rash 8%	10%
Mayer et al. [35], phase Ib, multicenter, open-label	**Alpelisib + Let** (300–350 + 2.5 mg/d)^c^	Postmenopausal HR+, HER2− mBC progressing on/after prior ET	26	Yes	*PIK3CA*-mutated, 25%; *PIK3CA*-wt, 10%	NA	NA	Alpelisib 300 mg/d Diarrhea 80% Nausea 60% Hyperglicemia 55% Rash 45% Fatigue 45%	Diarrhea 10% Hyperglicemia 10% AST/ALT elevation 5%	11%
Rugo et al. [51], phase 2, open-label, non-comparative study	**Alpelisib + Fulv**^**bd**^ (300 mg/d)^c^, **Alpelisib + Let**^**d**^ (300 + 2.5 mg/d)	Men and women with *PIK3CA*-mutated HR+, HER2− aBC whose disease progressed on/after CDK4/6i + ET	21, 18	Yes	20%, 18%	NA	NA	NA	Hyperglycemia 38.1% (Fulv)/27.8% (Let) Rash 4.8% (Fulv)/27.8% (Let)	5%, 5%

*AEs* adverse events, *AST* aspartate aminotransferase, *ALT* alanine aminotransferase, *CDKi* cyclin-dependent kinase inhibitors, *CT* chemotherapy, *d* day, *ET* endocrine therapy, *Fulv* fulvestrant, *G* grade, *mos* months, *HR* hormone receptor, *(L)ABC* (locally) advanced breast cancer, *mBC* metastatic breast cancer, *mg/d* milligrams per day, *mPFS* median progression-free survival, *mOS* median overall survival, *N*° number, *NA* not available, *NS* not specified, *ORR* overall response rate, *pts.* patients, *wt* wild type

^a^Study treatment discontinuation due to AEs

^b^Fulv 500 mg intramuscular injection on day 1, followed by 500 mg doses on days 15 and 28, and then every 28 days

^c^Refers to alpelisib

^d^Fulv cohort: patients treated with prior CDKi and aromatase inhibitors; Let cohort: patients treated with prior CDKi and Fulv

## Discussion

The recent registration of the Alp/Fulv combination for the treatment of *PIK3CA-*mutated HR+ HER2− mBC has been considered a biologically and clinically relevant advancement [10]. Indeed, Alp is the first compound that provided clinically meaningful benefit in a subgroup of HR+ HER2− mBC patients that can be identified on the basis of a specific genetic tumor biomarker.

Based on the comparison of efficacy and safety results of the BOLERO-2 and the SOLAR-1 studies, Eve or Alp in combination with standard ET provide similar PFS benefit when compared to ET alone; however, Alp/Fulv is associated with overall higher incidence of G3/G4 AEs despite the fact that patients in the SOLAR-1 trial had more favorable clinical characteristics, including better ECOG PS, absence of uncontrolled diabetes mellitus at enrollment, and the fact that metformin was administered as per protocol if fasting blood glucose concentration was 140 mg/dl or higher [36]. The toxicity profiles of Eve and Alp, which are partially non-overlapping, indicate that ongoing or future trials aiming to combine these compounds may result in exaggerated incidence of AEs, unless dosages of both drugs are reduced (NCT02077933).

Another crucial difference between Eve and Alp consists in the fact that patients with both *PIK3CA*-mutated and *PIK3CA*-wt tumors benefit from adding Eve to ET, while Alp selectively benefits patients with *PIK3CA-*mutated tumors, which account for approximately 40% of the HR+ HER2− BCs [16]. From a biological point of view, this is expected because Eve inhibits the PI3K/AKT/mTORC1 axis downstream of PI3K, i.e., independently of *PIK3CA* mutations or other PI3K/AKT/mTORC1 activating mechanisms. From a clinical point of view, this implies that Alp is not effective in about 60% of all HR+ HER2− mBC patients (i.e., those with *PIK3CA-*wt disease). On the other hand, Eve and Alp may provide similar relative PFS advantage in patients with *PIK3CA-*mutated neoplasms [30, 34]; however, this hypothesis derives from a NGS subanalysis of the BOLERO-2 trial and should be confirmed in prospective studies directly comparing Eve and Alp in patients with *PIK3CA*-mutated HR+ HER2− mBC.

Although indirect comparisons between independent trials cannot be used to draw definitive conclusions about different therapeutic approaches, and since no head-to-head trials can be expected soon, the available clinical evidence indicates that the “new” and more expensive Alp might be more toxic than the “old” Eve and has less broad clinical effectiveness (i.e., limited to patients with *PIK3CA-*mutated disease). For these reasons, the raising enthusiasm around Alp as a potential substitute of Eve in HR+ HER2− mBC treatment is not fully justified. The Eve/Exe combination remains a valid, and in many cases preferable (e.g., *PIK3CA-*wt neoplasms, or in diabetic or malnourished patients), treatment option for HR+ HER2− mBC patients undergoing disease progression on/after prior AI therapy.

Further clinical studies are needed to compare the efficacy and safety profile of Eve and Alp in HR+ HER2− mBC patients progressing on/after ET-CDK4/6 inhibitor treatment, which now represents the standard first- or second-line treatment in this clinical setting [2–4, 52–54]. In this clinical setting, the Eve/Exe combination and fulvestrant monotherapy remain two valid treatment options for patients with *PIK3CA-*wt neoplasms, while patients with *PIK3CA-*mutated neoplasms could potentially benefit from either fulvestrant/Alp or Eve/Exe. However, in the absence of clinical evidence, it is difficult to make clear clinical recommendations about the most effective second-line therapy in patients with *PIK3CA-*mutated or *PIK3CA-*wt tumors progressing on prior ET plus CDK 4/6 inhibitor-containing therapy. In patients with *PIK3CA-*mutated neoplasms, the sequential use of Eve and Alp in different treatment lines also deserves clinical investigation, at least in patients with *PIK3CA-*mutated disease; indeed, in the proof-of-concept phase III BELLE-3 trial, the pan-class I PI3K inhibitor buparlisib improved PFS when compared to the placebo in patients undergoing disease progression after prior Eve treatment, with an HR of 0.50 in the subgroup of *PIK3CA*-mutated neoplasms [55].

## Conclusions

When compared to the “old” everolimus, the “new” alpelisib may be burdened by higher incidence of severe adverse events, more narrow anticancer activity, and also higher costs after the approval of generic everolimus tablets (https://www.patient.novartisoncology.com/piqray-cost/; fda.gov/drugs/generic-drugs/overview-basics). The everolimus-exemestane combination remains an effective and reasonably well-tolerated second-line therapeutic option after progression to first-line AI plus/minus CDK 4/6 inhibitor treatment in HR+ HER2− mBC patients with *PIK3CA-*wt disease, as well as in patients with *PIK3CA-*mutated neoplasms who have contraindications to alpelisib, or those experiencing severe AEs during alpelisib/fulvestrant therapy.

## Data Availability

All data generated or analyzed during this study are included in this published article (and its supplementary information files).

## Abbreviations

AE: Adverse event
AI: Aromatase inhibitor
Alp: Alpelisib
AMPK: AMP-activated protein kinase
CDK 4/6: Cyclin-Dependent Kinase 4/6
CI: Confidence interval
ER: Estrogen receptor
ET: Endocrine therapy
Eve: Everolimus
FGFR1: Fibroblast growth factor receptor 1
Fulv: Fulvestrant
GPCR: G protein-coupled receptor
HER2: Human epidermal growth factor receptor 2
HR: Hazard ratio
HR+: Hormone receptor-positive
IGF-1: Insulin-like growth factor 1
INPP4B: Inositol polyphosphate 4-phosphatase type II (INPP4B)
IR: Insulin receptor
IRS1: Insulin receptor substrate 1
LKB1: Liver kinase B1
mBC: Metastatic breast cancer
mPFS: Median progression-free survival
mTORC1: Mammalian target of rapamycin complex 1
NSAI: Non-steroidal aromatase inhibitor
ORR: Overall response rate
OS: Overall survival
PI3K: Phosphoinositide 3-kinase
PIP2: Phosphatidylinositol 4,5-bisphosphate
PIP3: Phosphatidylinositol 3,4,5-triphosphate
PETN: Phosphatase and tensin homolog deleted from chromosome 10
S6K: S6 kinase
TSC 1/2: Tuberous sclerosis complex 1/2
4E-BP1: eIF4E-binding proteins
